# Physical and Sensory Long-Term Disabilities from *Bothrops* Snakebite Envenomings in Manaus, Western Brazilian Amazon

**DOI:** 10.3390/toxins17010022

**Published:** 2025-01-03

**Authors:** Eduardo M. G. Fernández, Débora N. Oliveira, Alexandre V. Silva-Neto, Rafaela N. Dávila, Ligia Lengler, Marco A. Sartim, Altair S. Farias, Luiz C. L. Ferreira, Érica da Silva Carvalho, Fan H. Wen, Felipe Murta, Fernando Almeida-Val, Manuela B. Pucca, Jacqueline A. G. Sachett, Wuelton M. Monteiro

**Affiliations:** 1 Graduate Program in Tropical Medicine, State University of Amazonas, Manaus 69040-000, Amazonas, Brazil; eduardomiguelgf@gmail.com (E.M.G.F.); nerydebs@gmail.com (D.N.O.); alexandre.silva@gmail.com (A.V.S.-N.); rafaelanunesdavila@gmail.com (R.N.D.); ligialengles@yahoo.com.br (L.L.); marcosartim@hotmail.com (M.A.S.); asfarias@uea.edu.br (A.S.F.); luiz.ferreira@gmail.com (L.C.L.F.); carvalhouea@gmail.com (É.d.S.C.); fan.wen@butantan.gov.br (F.H.W.); felipelmurta@gmail.com (F.M.); ffaval@gmail.com (F.A.-V.); jac.sachett@gmail.com (J.A.G.S.); 2 Dr. Heitor Vieira Dourado Foundation for Tropical Medicine, Manaus 69040-000, Amazonas, Brazil; 3 Graduate Program in Pharmaceutical Sciences, School of Pharmaceutical Sciences, Manaus 69040-000, Amazonas, Brazil; 4 Bioindustrial Center, Butantan Institute, São Paulo 05503-900, São Paulo, Brazil; 5 Department of Clinical Analysis, School of Pharmaceutical Sciences, São Paulo State University (UNESP), Araraquara 19060-900, São Paulo, Brazil

**Keywords:** snakebites, *Bothrops*, physical disability, sensory disability, public health

## Abstract

Snakebites caused by *Bothrops* snakes are the most prevalent in the Amazon region, causing local and systemic complications. Local complications are mostly represented by necrosis, secondary bacterial infection and compartment syndrome. There are reports of long-term disabilities, but their burden is poorly investigated. This study aims to describe and estimate the frequency of physical and sensory long-term disabilities from *Bothrops* snakebites in the Manaus Region, in the western Brazilian Amazon region. Participants were >18-years individuals that accepted to return to the hospital 3–12 months (average follow-up time of 195 days) after the discharge for neuromusculoskeletal, chronic pain and sensory assessments. Assessment of disability was also performed using the World Health Organization Disability Assessment Schedule 2.0 (WHODAS 2.0). Factors associated with summary disability using WHODAS 2.0 were identified. Fifty participants were enrolled. A frequency of 20% of the participants reported difficulty in moving the affected limb (20%), and 23.7% reported difficulty in walking. Limitations of daily activities were reported by 26% of the patients. Decreased strength of the affected limb was observed in 22% of the patients. Decreased range of joint motion was seen in 20% of the patients. Chronic pain was reported in 48% of the patients. Tactile sensibility was decreased in 30%, thermal sensibility in 14%, painful sensibility (hypoalgesia) in 12%, kinetic-postural sensibility (hypokinesthesia) in 4% and vibratory sensibility was decreased or abolished in 16% of the participants. Cognition and mobility domains were those with the highest frequencies of participants with any degree of disability, each with 57%. The summary WHODAS 2.0 disability rate was 59%. Age > 59 years (*p* = 0.02)] was associated with protection against disability. Difficulty in moving the limb (*p* = 0.05), pain at the affected limb (*p* < 0.01), limitations of daily activities (*p* < 0.01) and decreased thermal sensibility (*p* = 0.05) were significantly associated with disability. The present study consists of the first follow-up investigation involving *Bothrops* snakebite patients related to long-term disabilities. These findings represent important data on *Bothrops* snakebites causing clinically significant long-term neuromusculoskeletal and sensory disabilities, resulting in reduced quality of life of the patients.

## 1. Introduction

Snakebite envenomings (SBEs) occur when venom is injected through snake fangs, resulting in both local and systemic effects. According to the World Health Organization (WHO), 4.5 to 5.4 million people are bitten by snakes each year, with 1.8 to 2.7 million developing clinical symptoms and 81,000 to 138,000 dying from complications [1]. In Brazil, snakebites are particularly common, especially in the Amazon region. Data from the Notifiable Diseases Information System (SINAN) indicate that, by 2023, Brazil reported 32,420 cases of snakebites [2]. In the Amazon region, the mortality rate from SBEs stands at 1% in remote areas, considerably higher than the Brazilian average of 0.4% [3]. However, current epidemiological data likely underestimate the true scope of the problem, largely due to underreporting. This underreporting is driven by various factors, including the region’s limited access to healthcare services [4].

*Bothrops* venom is composed of a mixture of biological active components, responsible for diverse pathophysiological disturbs. The local effect of envenomation is characterized by intense inflammatory signs, such as pain and edema, that can be followed by bleeding and blood flow impairment, and is associated with complications, such as blister formation, necrosis and compartment syndrome [5]. The mechanisms rely on the action of important toxin classes, such as snake venom phospholipases A_2_ (SVPLA_2_) and metalloproteases (SVMP), among others, resulting in myotoxicity, skin tissue damage, vasculature disruption and nerve disturbs [6,7,8]. Considering that antivenom treatment presents some limited efficacy on local effects, *Bothrops* snakebite patients might present deficient regenerative outcomes, favoring long-term disabilities [9,10].

The extent of post-SBE complications lacks evidence, since considerably more attention has historically been given to acute manifestations [11]. Although studies have shown a rise in the number of cases, comprehensive research on the long-term complications of SBE remains limited [5,12]. Post-SBEs may lead to a series of long-lasting complications, with compartmental syndrome and amputation being the most severe outcomes [13]. Moreover, in addition to amputations, social stigma due to anxiety disorders, depression and post-traumatic disorders, psychological implications of pain or phantom limb sensation can also occur [14,15]. Other musculoskeletal complications such as arthritis, osteomyelitis, compartment syndrome, loss of muscle mass, muscle paralysis, Volkman’s contracture and Raynaud’s phenomenon are frequently reported [16,17,18]. In this study, we describe the clinical outcomes after several months of individuals who had an SBE and were managed in a reference hospital in Manaus, in the western Brazilian Amazon region.

## 2. Results

### 2.1. Epidemiological and Clinical Characteristics During Hospital Stay

Table 1 shows the demographic and clinical characteristics of the study participants. A total of 50 patients were evaluated in the study, comprising 38 men (76%) and 12 women (24%). The mean age of the participants was 49 years. Most snakebites occurred in rural areas. The feet were the most affected region, with 28 cases (56%). In terms of case severity, 10 patients (20%) were classified as mild, 24 (48%) as moderate and 16 (32%) as severe. The main local manifestations observed on admission were pain (100%), edema (90%) and ecchymosis (20%). Systemic manifestations included mostly headache (26%), nausea (12%), vomiting (6%) and systemic bleeding (4%). Regarding complications, secondary infection was the most prevalent, affecting 36% of the patients. Blood was incoagulable in the Lee–White test in 64% of the patients. A total of 32 patients (64%) were treated within 6 h, and 6 (12%) were treated after 24 h after the bite. The hospitalization stay was >8 days for 38% of the patients.

### 2.2. Neuromusculoskeletal and Chronic Pain Assessments

Table 2 shows the results from the neuromusculoskeletal assessment of the participants. A frequency of 20% of the patients reported difficulty moving the affected limb. In patients bitten in the lower limbs, 23.7% reported difficulty walking. Edema was observed in 18%, paresthesia in 12% and anatomical deformities in 10% of the patients. Chronic pain was reported in 48% of the patients, mostly moderate and intermittent. Generally, participants reported pain in the affected limb during movement, in some specific positions of the limb, during walking, when touching the affected limb, when the limb is hit or when wearing closed shoes. However, eight participants spontaneously reported pain “when it rains” and/or “when it is cold”. Three participants reported the pain as a puncture (“needle”, “pin” or “wasp stings”).

Limitations of daily activities caused by the snakebite were referred to by 26% of the patients. Scars were visible in 42% of the patients. Decreased strength of the affected limb was observed in 22% of the patients, in comparison with the contralateral limb. A decreased range of joint motion was seen in 20% of the patients. Hyporeflexia and hyperreflexia were present in 6% and 2% of the participants, respectively.

**Figure 1:**
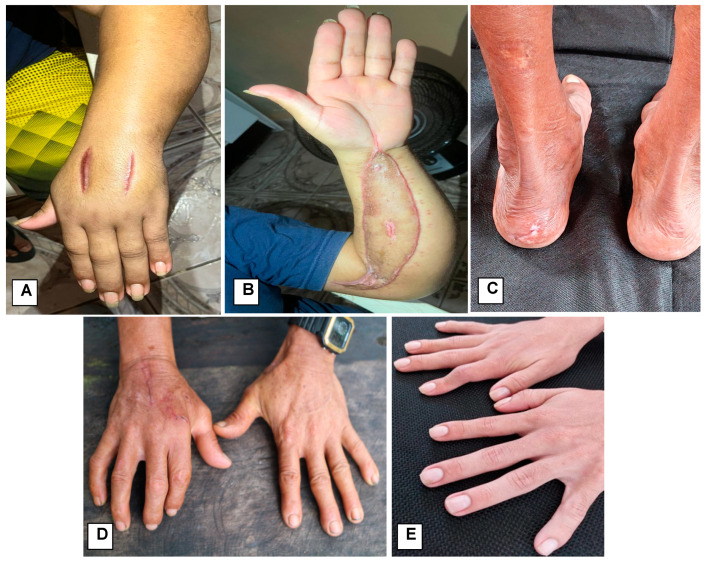
Photographs of patients presenting long-term disabilities. (**A**,**B**): A 29-year-old male patient bitten in the left hand, who received antivenom 5 h after the bite. Evolved with extensive compartment syndrome in the left upper limb, extending to the cervical region. It was evaluated by general surgeon who performed an emergency partial fasciotomy and indicated transfer for extensive fasciotomy. (**C**): A 69-year-old male patient bitten in the left heel, in his farm in the rural area of Manaus. On admission, he presented edema in left heel, moderate pain, perilesional paresthesia and local bleeding. He presented decreased tactile sensitivity in the heel area. (**D**): A 57-year-old male patient bitten in the back of his right hand. On admission, he had mild bruising on palmar region, edema at the wrist, hyperemia and local pain. He was treated with antivenom 1 h after the bite. He presented hypercromia of the affected area and decreased muscle strength. (**E**): A 24-year-old male patient bitten in the second phalanx region of the right hand when he was leaving the bathroom in the backyard of home, Manaus rural area. He was medically assisted about 1 h 30 min after the bite with complaints of pain, edema and bleeding at the bite site. On admission, he presented edema, hyperemia and intense pain up to the shoulder. He presented ankylosis of the middle phalanx of the finger as a late outcome.

Figure 1 shows some pictures of patients evolving with long-term complications.

### 2.3. Sensibility Assessments

Concerning long-term sensibility alterations, tactile sensibility was decreased in 30%, thermal sensibility in 14%, painful sensibility (hypoalgesia) in 12% and kinetic–postural sensibility (hypokinesthesia) in 4% of the patients. Vibratory sensibility was decreased in 14% and abolished in 2% of the participants (Table 3).

### 2.4. World Health Organization Disability Assessment Schedule 2.0 (WHODAS 2.0) Long-Term Disability Assessment

A total of 49 patients (1 abstained) included in the study completed the WHODAS 2.0 assessment. Cognition and mobility domains were those with the highest frequencies of participants with any degree of disability, each with 57%. The WHODAS 2.0 disability rate was 31% for self-care, 39% for getting along alone, 36% for life activities (domestic responsibilities), 38% for life activities (work) and 34% for participation. Severe disability ranged from 2% for participation to 16% for mobility. Extreme disability was observed only in the life activities (work) domain (2%). The summary WHODAS 2.0 disability rate was 59% (Table 4).

### 2.5. Factors Associated to WHODAS 2.0 Summary Disability

A total of 29 participants presented any disability (mild to extreme) by using the WHODAS 2.0 assessment tool. These were compared to 20 participants without disabilities.

Age > 59 years [OR = 0.1 (95%CI = 0.01–0.6); (*p* = 0.02)] was associated with the protection of any disability using WHODAS. The use of a tourniquet [OR = 4.7 (95%CI = 1.1–33.7); (*p* = 0.06)] and incoagulable blood on admission [OR = 3.3 (95%CI = 0.9–13.5); (*p* = 0.08)] were variables with a risk tendency for any disability (Table 5).

Difficulty in moving the limb [OR = 8.6 (95%CI = 1.4–165); (*p* = 0.05)], any pain at the affected limb [OR = 12.6 (95%CI = 2.9–54.1); (*p* < 0.01)], mild [OR = 9.3 (95%CI = 1.3–188); (*p* = 0.05)] or moderate [OR = 15.5 (95%CI = 2.5–305); (*p* = 0.01)] pain at the affected limb, limitations of daily activities after the snakebite [OR = 31.7 (95%CI = 1.7–576.3); (*p* < 0.01)], and decreased thermal sensibility [OR = 14.9 (95%CI = 0.8–276.2); (*p* = 0.05)] were significantly associated with the risk to any disability. Difficulty in walking [OR = 7.2 (95%CI = 1.2–141); (*p* = 0.07)] and chronic edema [OR = 7.2 (95%CI = 1.2–141); (*p* = 0.07)] were variables with a risk tendency for any disability (Table 6).

## 3. Discussion

Although the local effects of *Bothrops* envenomation are well-recognized, clinical findings regarding long-term complications remain primarily limited to case reports [19,20,21,22,23]. Regarding cases of *Bothrops* SBE in the Brazilian Amazon, these cases had in common a long delay in receiving antivenom treatment and evolved with serious local manifestations, such as secondary bacterial infections, extensive necrosis and compartment syndrome [13,20,21,22,23]. In a pioneering way, the present study presents results of a systematic investigation of key factors associated with *Bothrops* SBEs long-term disabilities in the region, which included neuromusculoskeletal, chronic pain and sensory function assessments.

The frequency of long-term disabilities from *Bothrops* SBEs was surprisingly high in our study, with at least 20% of the participants having discomfort moving the affected limb, difficulty in walking, decreased strength of the affected limb, a decreased range of joint motion and edema. Also noteworthy were the reports of chronic pain, by almost half of the participants, mostly moderate and intermittent. These disabilities resulted in limitations of daily activities reported by 26% of the patients. In our work, three (6%) of the patients had compartment syndrome, a condition that led to more severe long-term disabilities. In Nigeria, amputations from SBEs were associated with compartment syndrome in 19% of the cases [16]. In the Brazilian Amazon, compartment syndrome was the cause of amputation and limb atrophy in indigenous children [13]. In India, 19% of the patients still presented pain and 68% a low Patient-Specific Functional Scale score three months after discharge [24]; in agreement with our study, these authors also conclude that SBEs could be associated with some persisting functional disability even in the absence of local complications, such as skin necrosis and compartment syndrome. In Sri Lanka, musculoskeletal disorders, such as pain, local swelling, muscle weakness, deformities, contractures and amputations, were found in 3.2% [17]. Another study from the same country has shown that 4.4% of the patients had permanent musculoskeletal problems, none with a significant functional disability affecting daily routine [18]. Our findings indicate that male rural workers are most affected by SBE-related disabilities and, therefore, in addition to costs related to healthcare, with long hospital stays and expensive medications, a very large financial impact is also expected for family subsistence and the productivity loss from a societal perspective [25].

Chronic pain was the most common long-term manifestation in this study and was reported in 48% of the patients. Most of these patients reported that the pain was moderate and intermittent, and appeared while moving the limb and carrying out daily activities. Moreover, chronic pain, even mild to moderate, was strongly associated with global WHODAS 2.0 disability. Chronic pain is associated with behavioral health conditions, including mental disorders, substance use disorders and an increased risk of suicidal ideation [26,27,28], cognitive impairment [29], sleep disturbances [30] and fall-related injuries in older adults [31]. The frequency, limitations in activities, lost work productivity, reduced quality of life and potentially harmful outcomes associated with chronic pain from *Bothrops* SBEs underscore the importance of adequately treating and providing care to these patients. To achieve this aim, it is important to consider the pharmacologic and nonpharmacologic treatments for pain care, which are accessible to patients with pain and their treating clinicians [32]. Rural Amazonian populations still resort to indigenous medicine for pain relief, in combination with western medicine, mostly not prescribed by clinicians, demonstrative of an advanced stage of medical pluralism, especially in villages that are located closer to urban areas [33,34]. The dialogue between theses medicines is critical for the provision of culturally competent care for SBE patients with chronic pain.

Chronic edema was reported in 18% of the patients and was significantly associated with global WHODAS 2.0 disability. Chronic lymphedema caused by *B. atrox* was previously reported in an elder male patient, after 5 years of envenomation, with extensive edema and skin thickening accompanied by pain, paresthesia and itching in the affected limb [5]. Tissue damage caused directly by the toxins or by microvascular alterations and secondary bacterial infection can cause changes in the venous and lymphatic system conducive to chronic edema [35,36]. Chronic edema of the lower limbs is a debilitating and progressive condition that can cause daily difficulties, life-long physical, psychological and social problems [37,38,39]. Compression-wrapping devices are indicated for the management of chronic edema [40], but none of the patients were using these devices.

Long-term sensory impairment was observed in the affected limb of a third of the participants, especially the decrease of tactile, thermal and painful sensibilities. Chronic sensory neuropathy was not properly investigated, but it is probably related to metabolic changes because of ischemia, the inflammatory process and secondary bacterial infection, which can result in nerve injury [41]. *Bothrops* toxins can induce neuromuscular blockade associated with nerve degeneration, with Schwann cells damage, edematous axons and mitochondria and myelin sheath alteration [42]. Sensory neuropathy, mostly caused by diabetes, results in the loss of protective sensation allowing injury to go unnoticed, and loss of the normally subtle protective changes in biomechanical load distribution, which can result in sustained stress and tissue damage [43]. In the case of *Bothrops* SBEs, which mainly occurs in young adult individuals, the patient will live with the problem for a long time, and the consequences for their health are potentially serious. An additional and intriguing finding was that eight participants spontaneously reported experiencing pain “when it rains” and/or “when it is cold”. Recently, Raynaud’s phenomenon, characterized by cold-induced vasospastic disorders, was reported two months after snakebite envenoming by Nikolsky’s viper [44]. Although this phenomenon has not been previously described in cases of *Bothrops* envenoming, the cold-related sensations reported by the participants warrant further investigation to explore potential similarities or underlying mechanisms.

The WHODAS 2.0 disability assessment showed losses in all domains, with a greater impact on cognition and mobility, which were reduced in most patients. The cognition domain involves concentration, memory, understanding and communicating, and could be caused by injury to the central nervous system resulting from venom-induced consumption coagulopathy, which leads to ischemic and hemorrhagic phenomena [22]. In addition to CNS injury, anxiety, depression and post-traumatic disorders and psychological implications of chronic pain may trigger cognitive impairment [14,15]. Long-term sensory impairment is also associated with cognitive impairment [45]. Mobility problems also contributed greatly to the high disability frequency detected by the WHODAS 2.0 tool and is a consequence of the musculoskeletal sequelae described above. Self-care, interacting with other people, life activities (domestic responsibilities, leisure, work and school activities) and participation domains may be impaired by both neuropsychological and motor disorders. Large scars, edema, anatomical deformities and altered limb movement can result in stigma and social rejection, which is a major barrier to participation described in several neglected tropical diseases [46].

Unexpectedly, older age was associated with being a protection for the global disability detected by WHODAS 2.0. We believe that this association is due to a confounding bias, since this age group already has a functional decline caused by ageing and comorbidities, both cognitive and physical, as well as reduced social participation compared to younger adult people. For instance, an event such as a snakebite may have a less noticeable impact on a patient who already faces progressive deterioration in sensory function resulting from aging [47,48]. Difficulty in moving the limb, chronic pain at the affected limb, self-reported limitations of the daily activities after the snakebite, decreased thermal sensibility and chronic edema were significantly associated with the risk of global disability detected by WHODAS 2.0. Use of tourniquet before hospital admission and incoagulable blood on admission were also variables with a risk tendency for long-term disability. The use of tourniquet was statistically associated with the presence of necrosis in *Bothrops jararaca* SBEs [49], and eventually with chronic neuromuscular disorders. Incoagulable blood on admission was associated with bleeding disorders [49] and poor outcomes [50] in *Bothrops* SBEs in Brazil.

One potential source of bias in our research is that participants who agreed to take part were predominantly those who experienced the most severe sequelae, which could introduce bias during sampling.

## 4. Conclusions

Snakebites caused by *Bothrops* causes clinically relevant long-term neuromuscular disabilities, chronic pain and sensory impairment, which result in a reduced quality of life in patients. Disabilities resulting from SBEs were less noticeable in older patients, possibly due to concurrent ageing-related functional loss. Difficulty in moving the bitten limb, chronic pain, limitations in daily activities after the snakebite and decreased thermal sensibility were significantly associated with any degree of disability detected by WHODAS 2.0. Early rehabilitative support is potentially beneficial for this group of patients. Further research should concentrate on the burden of SBE-related long-term morbidity in a larger cohort of patients and on developing effective interventions.

## 5. Materials and Methods

### 5.1. Study Design and Population

This is a case series study focusing on patients who have experienced *Bothrops* SBEs and who were treated at the Fundação de Medicina Tropical Dr. Heitor Vieira Dourado (FMT-HVD) in the State of Amazonas, Brazil. Eligible participants included >18-years individuals with confirmed SBEs. Exclusion criteria were defined as any patient unwilling to return to the hospital for functionality assessments or unable to provide informed consent.

### 5.2. Hospital Management of the SBEs

Patients with confirmed *Bothrops* SBEs on hospital admission were treated with *Bothrops* or *Bothrops-Lachesis* antivenom according to the guidelines of the Brazilian Ministry of Health, and cases were classified as (1) mild: local pain, swelling and bruising; (2) moderate: local manifestations without necrosis and minor systemic signs (coagulopathy and bleeding, no shock); or (3) severe: life-threatening snakebite, with severe bleeding, hypotension, shock and/or acute kidney failure [51]. Mild cases were treated with 2–4 antivenom vials, moderate cases with 4–8 vials and severe cases with 12 vials. On admission, information was collected on age, gender, marital status, place of residence (rural or urban), anatomical site of the bite, comorbidities, time from bite to admission, use of tourniquet and clotting time using the Lee–White test. Local and systemic clinical manifestations were collected over the hospital stay. The affected limb was treated in the most comfortable position, according to patient preferences. When present, blisters were aspirated, necrotic tissue was surgically debrided, abscesses were drained and antibiotic treatment was given accordingly.

### 5.3. Long-Term Functionality Assessment

Eligible patients were invited to return to the hospital 3–12 months after discharge. Patients were contacted by a phone call, and those who attended the call and accepted to return were assessed for functionality.

#### 5.3.1. Neuromusculoskeletal and Pain Assessments

Participants underwent a comprehensive clinical assessment to evaluate their physical condition, which included:

- Physical examination to detect difficulty moving the limb, difficulty walking, edema, scars, anatomical deformities, paresthesia and other alterations;
- Self-reported limitations of daily activities after the snakebite;
- Pain assessment using a numerical rating scale, with values ranging from 1 to 10 [52]. Pain was further classified as absent (rate 0), mild (rated from 1 to 3), moderate (rated from 4 to 7) and intense (rated from 8 to 10). Pain was also classified as intermittent or continuous;
- Muscle strength: Measured using manual muscle testing methods to determine the strength of the affected and its contralateral limb [53]. Muscle strength was scored according to the MRC scale, and further classified as normal or decreased and on whether the difference between the affected and contralateral limb was >2;
- Range of joint movement: Evaluated with a goniometer to assess the mobility of joints of the affected limb [54]. The test result was classified as normal or decreased and on whether the difference in the range of movement between the affected and contralateral limb was >20°;
- Deep osteotendinous reflexes: Tested using a reflex hammer to evaluate the integrity of the neuromuscular system. Test result was classified as normal or decreased.

#### 5.3.2. Sensibility Assessments

Sensory evaluations included:

- Tactile sensibility: Evaluated with an esthesiometer applied to the affected segment, comparing it with the same region of the contralateral limb. Test result was classified as normal or decreased;
- Thermal sensibility: Evaluated with cold (5–10 °C) and hot water (40–45 °C) tubes applied to the affected segment, comparing it with the same region of the contralateral limb. Test result was classified as normal or decreased;
- Painful sensibility: Evaluated with a number 11 crochet needle applied to the affected segment, comparing it with the same region of the contralateral limb. Test result was classified as normal or decreased;
- Kinetic–postural sensibility: Evaluated by moving the affected limb segment in various directions, stabilizing the segment at a given moment in a position, which must be recognized by the patient. Test result was given as normal, akinesthesia or hypokinesthesia;
- Vibratory sensibilities: Tested using a tuning fork (128 or 258 Hz), placing it on the bone protrusions chosen depending on the affected area, and comparing with the contralateral region. Test result was classified as apalesthesia, hypopalesthesia or normopalesthesia.

#### 5.3.3. WHODAS 2.0 Long-Term Functionality

We used the 36-item WHODAS 2.0 questionnaire, which was administered by interviewing the participants. Participants were asked to consider how much their disabilities interfered with their lives and then they answered on a 5-point response scale from 1 (none) to 5 (extreme/cannot do). The scoring algorithm that we used is available through the WHO. The WHODAS 2.0 consists of six domains: 1. Cognition (understanding and communicating), 2. Mobility (moving and getting around), 3. Self-care (hygiene, dressing, eating and staying alone), 4. Getting along (interacting with other people), 5. Life activities (5.1. Domestic responsibilities and 5.2. Work) and 6. Participation (joining in community activities). A summary score (the general-disability latent variable) was also obtained. All scores ranged from 0 (best) to 100 (worst), and high scores indicated a high level of disability. For each domain and for summary disability, the frequency of participants with no, mild, moderate, severe and extreme disability was calculated [55].

### 5.4. Data Analysis

Data were entered into REDCap (Vanderbil University, USA). The comparison of the frequency of clinic-epidemiological variables during the hospital stay and functionality parameters between the participants presenting any disability (mild to extreme) was obtained by using WHODAS 2.0. For those without disabilities, the comparison was made using the χ^2^ or Fisher exact test; odds ratios (ORs) with 95% CIs were obtained. Statistical analyses were performed in the R software in the IDE RStudio environment, version 4.1.2 (Posit PBC), and the significance level of the tests was *p* < 0.05.

## Figures and Tables

**Table 1:** Demographic and clinical characteristics of the study participants on admission and during hospitalization stay (n = 50).

Characteristic	Frequency (n = 50)
Gender	
Male	38 (76%)
Female	12 (24%)
Age (mean/±standard deviation)	49 (±15)
Age groups (years)	
18–39	14 (28%)
40–59	25 (50%)
>59	11 (22%)
Marital status ^1^	
Single	11 (37.9%)
Married	18 (62.1%)
Area of the residence	
Rural	42 (84%)
Urban	8 (16%)
Affected region	
Foot	31 (62%)
Hand	11 (22%)
Leg	7 (14%)
Arm	1 (2%)
Use of tourniquet	12 (24%)
Case severity on admission ^2^	
Mild	10 (20%)
Moderate	24 (48%)
Severe	16 (32%)
Local manifestations on admission	
Pain	50 (100%)
Edema	45 (90%)
Ecchymosis	10 (20%)
Bleeding	9 (18%)
Blisters	9 (18%)
Necrosis	1 (2%)
Systemic manifestations on admission	
Headache	13 (26%)
Nausea	6 (12%)
Vomiting	3 (6%)
Gum bleeding	1 (2%)
Hematemesis	1 (2%)
Snakebite complications during hospitalization	
Secondary bacterial infection	18 (36%)
Acute kidney injury	4 (8%)
Necrosis	3 (6%)
Compartment syndrome	3 (6%)
Comorbidities ^3^	
Yes	11 (22%)
Lee–White Clotting Time	
Normal	18 (36%)
Incoagulable (in 10 min)	32 (64%)
Time until medical care (hours)	
<6	36 (72%)
6–12	6 (12%)
12–24	2 (4%)
>24	6 (12%)
Hospitalization stay (in days)	
<4	19 (38%)
4–8	12 (24%)
>8	19 (38%)

^1^ Information available for 29 individuals. ^2^ According the Brazilian Ministry of Health guideline. ^3^ In descending order: systemic arterial hypertension (n = 9), diabetes mellitus (n = 1), congestive heart failure (n = 1), poliomyelitis-related disability (n = 1), osteoarthritis (n = 1), labyrinthitis (n = 1) and ankylosing spondylitis (n = 1).

**Table 2:** Long-term findings of the neuromusculoskeletal assessment of the study participants (n = 50).

Variables	Frequency (n = 50)
Difficulty moving the limb	10 (20%)
Difficulty walking (n = 38) ^1^	9 (23.7%)
Edema	9 (18%)
Paresthesia	6 (12%)
Anatomical deformities	5 (10%)
Tremors	2 (4%)
Pain at the affected limb	
Yes	24 (48%)
Pain intensity ^2^	
Absent	26 (52%)
Mild	7 (14%)
Moderate	14 (28%)
Intense	3 (6%)
Pain characteristic ^3^	
Intermittent	21 (87.5%)
Continuous	3 (12.5%)
Limitations of daily activities caused by the snakebite	13 (26%)
Presence of scar	21 (42%)
Type of scar	
According to the color	
Normochromic	16 (76.2%)
Hyperchromic	4 (19%)
Hypochromic	1 (4.8%)
According to the morphology	
Normotrophic	11 (52.4%)
Hypertrophic	6 (28.6%)
Retracted	3 (14.3%)
Atrophic	1 (4.8%)
Fasciotomy scar	3 (6%)
Strength of the affected limb ^4^	
Normal	46 (93.9%)
Decreased	3 (6.1%)
Range of joint motion ^4^ N = 49	
Normal	42 (85.7%)
Decreased	7 (14.3%)
Deep osteotendinous reflexes	
Normal	46 (92%)
Hyporeflexia	3 (6%)
Hyperreflexia	1 (2%)

^1^ Cases in the lower limbs in the denominator. ^2^ Pain assessment was made using a numerical rating scale, with values ranging from 1 to 10; pain was further classified as absent (rate 0), mild (1 to 3), moderate (4 to 7) or intense (8 to 10). ^3^ Cases presenting chronic pain in the denominator. ^4^ Compared to the contralateral limb.

**Table 3:** Long-term sensibility alterations in the affected limb of the study participants (n = 50).

Variables	Frequency (n = 50)
Tactile sensibility	
Normal	35 (70%)
Decreased	15 (30%)
Thermal sensibility	
Normal	42 (86%)
Decreased	8 (14%)
Painful sensibility	
Normal	42 (88%)
Hypoalgesia	8 (12%)
Kinetic–postural sensibility	
Normal	48 (96%)
Hypokinesthesia	2 (4%)
Vibratory sensibility	
Normal	42 (84%)
Hypoesthesia	7 (14%)
Apalesthesia	1 (2%)

**Table 4:** Distribution of disability grades assessed by WHODAS 2.0 (n = 49).

Domain	Disability Grade by Frequency				
	Absent	Mild	Moderate	Severe	Extreme
1. Cognition	43%	26%	23%	8%	0%
2. Mobility	43%	25%	16%	16%	0%
3. Self-care	69%	21%	6%	4%	0%
4. Getting along	61%	21%	14%	4%	0%
5.1 Life activities (Domestic responsibilities)	64%	12%	16%	8%	0%
5.2 Life activities (Work)	62%	12%	12%	12%	2%
6. Participation	66%	18%	14%	2%	0%
Summary disability	41%	39%	16%	4%	0%

**Table 5:** Association between epidemiological and clinical factors on hospital admission and Summary Disability Assessment detected by WHODAS 2.0 (n = 49).

Variable	No Disability (n = 20)	Mild to Severe Disability (n = 29)	Odds Ratio (95%CI)	*p*
Gender				
Male	18 (90.0%)	20 (69.0%)		
Female	2 (10.0%)	9 (31.0%)	4.1 (0.89–28.9)	0.10
Age groups (years)				
18–39	2 (10.0%)	11 (37.9%)		
40–59	11 (55.0%)	14 (48.3%)	0.2 (0.03–1.1)	0.09
>59	7 (35.0%)	4 (13.8%)	0.1 (0.01–0.6)	0.02
Marital status ^1^				
Single	2 (25.0%)	9 (42.9%)	—	
Married	6 (75.0%)	12 (57.1%)	0.4 (0.1–2.5)	0.40
Area of the residence				
Rural	16 (80.0%)	26 (89.7%)		
Urban	4 (20.0%)	3 (10.3%)	0.5 (0.1–2.4)	0.30
Affected region				
Lower limbs	13 (65.0%)	25 (86.2%)		
Upper limbs	7 (35.0%)	4 (13.8%)	0.3 (0.1–1.2)	0.16
Use of tourniquet	2 (10.0%)	10 (34.5%)	4.7 (1.1–33.7)	0.06
Case severity on admission ^2^				
Mild	4 (20.0%)	6 (20.7%)		
Moderate	13 (65.0%)	10 (34.5%)	0.5 (0.1–2.3)	0.40
Severe	3 (15.0%)	13 (44.8%)	2.9 (0.5–19.0)	0.20
Local manifestations on admission				
Pain	20 (100.0%)	29 (100.0%)		>0.99
Edema	18 (90.0%)	26 (89.7%)	0.9 (0.12–6.4)	>0.99
Ecchymosis	5 (25.0%)	5 (17.2%)	0.6 (0.15–2.6)	0.50
Bleeding	5 (25.0%)	4 (13.8%)	0.5 (0.10–2.1)	0.30
Blisters	2 (10.0%)	7 (24.1%)	2.9 (0.60–20.9)	0.20
Lymph node enlargement	1 (5.0%)	3 (10.3%)	2.2 (0.26–46.2)	0.50
Necrosis	0 (0.0%)	1 (3.5%)	1.4 (0.04–43.6)	>0.99
Systemic manifestations on admission				
Headache	6 (30.0%)	7 (24.1%)	0.7 (0.2–2.7)	0.60
Nausea	2 (10.0%)	4 (13.8%)	1.4 (0.3–11.2)	0.70
Vomiting	1 (5.0%)	2 (6.9%)	1.4 (0.1–31.6)	0.80
Gum bleeding	0 (0.0%)	1 (3.5%)	1.4 (0.04–43.6)	>0.99
Hematemesis	0 (0.0%)	1 (3.5%)	1.4 (0.04–43.6)	>0.99
Complications during hospitalization				
Secondary bacterial infection	6 (30.0%)	12 (41.4%)	1.6 (0.5–5.8)	0.40
Acute kidney injury	3 (15.0%)	1 (3.5%)	0.2 (0.01–1.7)	0.20
Necrosis	0 (0.0%)	3 (10.4%)	4.5 (0.04–43.6)	0.66
Compartment syndrome	0 (0.0%)	3 (10.4%)	4.5 (0.04–43.6)	0.66
Comorbidities ^3^				
Yes	2 (10.0%)	9 (31.0%)	0.3 (0.05–1.3)	0.16
Lee–White Clotting Time				
Normal	16 (80.0%)	16 (55.2%)		
Incoagulable (in 10 min)	4 (20.0%)	13 (44.8%)	3.3 (0.9–13.5)	0.08
Time until hospital admission (hours)				
<6	16 (80.0%)	20 (69.0%)		
6–12	3 (15.0%)	3 (10.3%)	0.8 (0.1–4.8)	0.80
12–24	1 (5.0%)	1 (3.5%)	0.8 (0.03–21.3)	0.90
>24	0 (0.0%)	5 (17.2%)	7.8 (0.4–152.8)	0.28

^1^ Information available for 29 individuals. ^2^ According the Brazilian Ministry of Health guideline. ^3^ In descending order: systemic arterial hypertension (n = 9), diabetes mellitus (n = 1), congestive heart failure (n = 1), poliomyelitis-related disability (n = 1), osteoarthritis (n = 1), labyrinthitis (n = 1) and ankylosing spondylitis (n = 1).

**Table 6:** Association between long-term complications and Summary Disability Assessment detected by WHODAS 2.0 (n = 49).

Variable	No Disability (n = 20)	Mild to Severe Disability (n = 29)	Odds Ratio (95%CI)	*p*
Difficulty moving the limb	1 (5.0%)	9 (31.0%)	8.6 (1.4–165)	0.05
Difficulty walking (n = 38) ^1^	1 (5.0%)	8 (27.6%)	7.2 (1.2–141)	0.07
Edema	1 (5.0%)	8 (27.6%)	7.2 (1.17–141)	0.07
Anatomical deformities	1 (5.0%)	4 (13.8%)	3.0 (0.41–62.1)	0.30
Pain at the affected limb				
Yes	3 (15.0%)	20 (69.0%)	12.6 (2.9–54.1)	<0.01
Pain intensity ^2^				
Absent	16 (80.0%)	10 (34.5%)		
Mild	1 (5.0%)	6 (20.7%)	9.3 (1.3–188)	0.05
Moderate	2 (10.0%)	11 (37.9%)	15.5 (2.5–305)	0.01
Intense	1 (5.0%)	2 (6.9%)	3.1 (0.3–71.4)	0.40
Limitations of the daily activities after the snakebite	0 (0.0%)	13 (44.8%)	31.7 (1.7–576.3)	<0.01
Presence of scar	6 (30.0%)	14 (48.3%)	2.2 (0.7–7.6)	0.20
Strength of the affected limb				
Normal	19 (95.0%)	27 (93.1%)		
Decreased	1 (5.0%)	2 (6.9%)	1.4 (0.1–31.6)	0.80
Range of joint motion ^3^				
Normal	17 (85.0%)	24 (82.8%)		
Decreased	3 (15.0%)	5 (17.2%)	1.2 (0.3–6.4)	0.80
Deep osteotendinous reflexes				
Normal	20 (100.0%)	25 (86.2%)		
Hyporeflexia	0 (0.0%)	3 (10.3%)	4.7 (0.2–98.9)	0.64
Hyperreflexia	0 (0.0%)	1 (3.5%)	1.6 (0.05–48.9)	>0.99
Tactile sensibility				
Normal	16 (80.0%)	19 (65.5%)		
Decreased	4 (20.0%)	10 (34.5%)	2.1 (0.6–8.8)	0.30
Thermal sensibility				
Normal	20 (100.0%)	21 (72.4%)		
Decreased	0 (0.0%)	8 (27.6%)	14.9 (0.8–276.2)	0.05
Painful sensibility				
Normal	18 (90.0%)	23 (79.3%)		
Hypoalgesia	2 (10.0%)	6 (20.7%)	2.4 (0.5–17.4)	0.30
Kinetic–postural sensibility				
Normal	20 (100.0%)	27 (93.1%)		
Hypokinesthesia	0 (0.0%)	2 (6.9%)	2.9 (0.1–67.6)	>0.99
Vibratory sensibility				
Normal	17 (85.0%)	24 (82.8%)		
Hypoesthesia	3 (15.0%)	4 (13.8%)	0.9 (0.2–5.0)	0.90
Apalesthesia	0 (0.0%)	1 (3.5%)	1.4 (0.04–43.6)	>0.99
Time from bite to functionality assessment (months) ^4^				
3–6	11 (55.0%)	17 (58.7%)		
7–9	6 (30.0%)	7 (24.1%)	1.3 (0.4–4.9)	0.67
10–12	3 (15.0%)	5 (17.2%)	0.9 (0.2–4.7)	>0.99

^1^ Cases in the lower limbs in the denominator. ^2^ Pain assessment was made using a numerical rating scale, with values ranging from 1 to 10; pain was further classified as absent (rate 0), mild (1 to 3), moderate (4 to 7) or intense (8 to 10). ^3^ Compared to the contralateral limb. ^4^ Average follow-up time was 195 days.

## Data Availability

The raw data supporting the conclusions of this article will be made available by the authors on request.
